# Unravelling the Genetic History of Negritos and Indigenous Populations of Southeast Asia

**DOI:** 10.1093/gbe/evv065

**Published:** 2015-04-14

**Authors:** Farhang Aghakhanian, Yushima Yunus, Rakesh Naidu, Timothy Jinam, Andrea Manica, Boon Peng Hoh, Maude E. Phipps

**Affiliations:** ^1^Jeffrey Cheah School of Medicine and Health Sciences, Monash University (Malaysia), Selangor, Malaysia; ^2^Institute of Medical Molecular Biotechnology, Faculty of Medicine, Universiti Teknologi MARA, Selangor, Malaysia; ^3^Division of Population Genetics, National Institute of Genetics, Mishima, Japan; ^4^Evolutionary Ecology Group, Department of Zoology, University of Cambridge, United Kingdom

**Keywords:** Negritos, Senoi, Proto-Malay, population genetics, SNPs

## Abstract

Indigenous populations of Malaysia known as Orang Asli (OA) show huge morphological, anthropological, and linguistic diversity. However, the genetic history of these populations remained obscure. We performed a high-density array genotyping using over 2 million single nucleotide polymorphisms in three major groups of Negrito, Senoi, and Proto-Malay. Structural analyses indicated that although all OA groups are genetically closest to East Asian (EA) populations, they are substantially distinct. We identified a genetic affinity between Andamanese and Malaysian Negritos which may suggest an ancient link between these two groups. We also showed that Senoi and Proto-Malay may be admixtures between Negrito and EA populations. Formal admixture tests provided evidence of gene flow between Austro-Asiatic-speaking OAs and populations from Southeast Asia (SEA) and South China which suggest a widespread presence of these people in SEA before Austronesian expansion. Elevated linkage disequilibrium (LD) and enriched homozygosity found in OAs reflect isolation and bottlenecks experienced. Estimates based on *N*_e_ and LD indicated that these populations diverged from East Asians during the late Pleistocene (14.5 to 8 KYA). The continuum in divergence time from Negritos to Senoi and Proto-Malay in combination with ancestral markers provides evidences of multiple waves of migration into SEA starting with the first Out-of-Africa dispersals followed by Early Train and subsequent Austronesian expansions.

## Introduction

The events and period of prehistoric peopling of Southeast Asia (SEA) have been controversial. Human remains from archeological sites such as Callao Cave in Philippines (Mijares et al. 2010) and Niah Cave in Malaysia (Barker et al. 2007) suggest that SEA was populated by anatomically modern humans approximately 50–70 kilo years ago (KYA). In 2009, a large-scale genome-wide study by the HUGO-Pan Asia consortium showed that all East Asians and Southeast Asians originated from a single wave “Out-of-Africa” via a southern coastal route (HUGO Pan-Asia SNP Consortium 2009). Thereafter, two models have been proposed to explain subsequent migrations involved in shaping todays SEA populations. The Out-of-Taiwan model refers to the Austronesian language expansion that occurred around 5,000–7,000 years before the present. This replaced the pre-existing Australoid people with Austronesian agriculturists (Diamond and Bellwood 2003; Bellwood 2005). In the long period between the first initial Out-of- Africa and the recent “Out-of-Taiwan” migrations, recent genetic studies on mitochondrial DNA (mtDNA) suggest an Early Train wave of migration during the late Pleistocene to early Holocene (Hill et al. 2006, 2007; Soares et al. 2008; Karafet et al. 2010; Jinam et al. 2012).

The rich ethnological diversity that exists in Peninsular Malaysia provides a great opportunity to study SEA prehistory. The current Malaysian population comprises three major ethnic groups including Malay, Chinese, and Indians. In addition to these groups, Peninsular Malaysia is home to other ethnicities including several minor indigenous communities collectively known as “Orang Asli” (OA) or “Original People.” Making up approximately 0.6% of Malaysian population, OA has been classified into three groups, namely Negrito (Semang), Senoi, and Proto-Malay (aboriginal Malay) based on linguistic, physical, and anthropological characteristics. Each OA group could be further subdivided into six subgroups based on their lifestyle and geographical location.

Malaysian Negritos are Austro-Asiatic (AA) speakers and inhabit in northern parts of Peninsular Malaysia. The tradition of these hunter-gatherers involves northern Aslian dialect of AA language, egalitarianism, and patrilineal descent system. On the basis of their hunter-gathering lifestyle and physical characteristics including their small body size, dark skin pigmentation, cranio-facial morphology, and frizzy hair, Malaysian Negritos traditionally are grouped with other Negrito communities in South Asia and SEA such as Andaman islanders, Mani in Thailand, Philippine Negritos, and other phenotypically similar populations in Papua New Guinea and Australia. These similarities have led to the general idea that all Negrito populations of SEA and Oceania originated from a common ancestral group which entered SEA during the earliest human dispersals into Asia (Endicott 2013). However, genetic studies have provided mixed evidence. Although a genetic affinity between Andaman islanders, Malaysian and Philippine Negritos was detected by some authors (Jinam et al. 2012; Chaubey and Endicott 2013), several mtDNA (Endicott et al. 2003; Thangaraj et al. 2005; Wang et al. 2011), Y chromosome (Delfin et al. 2011; Scholes et al. 2011), and autosomal (HUGO Pan-Asia SNP Consortium 2009) studies indicate that Negrito populations are closer to their neighboring non-Negrito communities.

Senoi, who are AA speakers, make up the largest group among the OA populations. They traditionally practice slash-and-burn farming and their phenotypic features are intermediate between Australoid and Mongoloid people. The origin of the Senoi is obscure; however, based on archeological and limited genetic studies, they have been linked with AA agriculturists from mainland SEA or South China who arrived in Peninsular Malaysia in the mid-Holocene (Hill et al. 2006). Proto-Malays exhibit Mongoloid feature and speak Austronesian dialects. They are taller, fairer, and may have straighter hair. These are the agriculturists and fishermen who are believed to have settled in coastal areas of Malaysia during the Austronesian (out-of-Taiwan) expansion.

Previous studies of these Malaysian populations have relied on relatively small sample sizes and low density genetic markers, limiting the power of the analysis. Here, we provide a more comprehensive insight and better estimate of divergence time for populations in SEA, by leveraging on larger sample sizes on very high-density Illumina HumanOmni 2.5 BeadChip arrays. We first investigated how distinct OAs are from other Asian populations, quantifying genetic structure within the Asian continent. We also examined linkage disequilibrium (LD) decay and runs of homozygosity (ROH) to study population history and consanguinity. Finally, we examined gene flow between OA population and other populations in East Asian (EA) and estimated the divergence time for these populations to elucidate events involved in the peopling of SEA.

## Materials and Methods

### Ethics Statements, Sample Collection, and Genotyping

This study was approved by the Ministry of Health Malaysia under National Medical Research Registry MNDR ID #09—23-3913, JAKOA (Department of Orang Asli Development, Government of Malaysia) and Monash University Human Research Ethics Committee.

Following consultation with JAKOA officers in the various districts in different states, courtesy visits were made to OA community elders and the rationale of the study and the procedure of sample collection explained. Once they had agreed and informed their communities, field visits were carried out. Individuals who provided informed consent and also answered questionnaires were included.

**Fig. 1.—:**
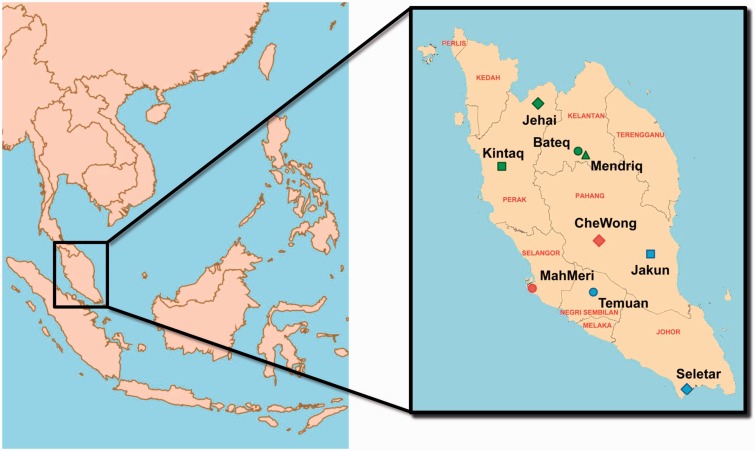
Geographical location of Orang Asli communities recruited in this study.

Peripheral blood samples were collected from 169 individuals belonging to Negrito (Jehai, Bateq, Kintaq, and Mendriq subgroups), Senoi (MahMeri and CheWong subgroup), and Proto-Malay (Seletar, Jakun, and Temuan subgroups) groups (fig. 1). Genotyping was performed using Illumina Human Omni 2.5 array (Illumina Inc., San Diego, CA).

### Quality Control and Data Integration

Quality controls were applied to the data obtained from each OA community separately to exclude problematic samples and single nucleotide polymorphisms (SNPs). All SNPs that failed the Hardy–Weinberg exact (HWE) test (*P* < 10^−^^6)^ and displayed missing rates >0.05 across all samples in each population were removed. Additionally, samples with call rate <0.99 were excluded. Gender concordance was examined using PLINK v1.07 (Purcell et al. 2007) and samples with inconsistency between genotype results and questionnaire-reported sex were excluded. In order to avoid analysis of close relatives, unknown relatedness was measured between all pairs of individuals within each population using PLINK’s (v1.07) Identity-by-Descent estimation, PI_Hat. An upper cut-off threshold of 0.375 was set to exclude first-degree relatedness within each population. Finally, a principal component analysis (PCA) using EIGENSOFT v3.0 (Patterson et al. 2006) was performed to remove outliers from each population across first ten eigenvectors. In the final stage, all OA populations were merged into one data set and pruned for SNPs that failed HWE (*P* < 10^−^^6) test^ and missing rates more than 0.05 across all samples.

The OA genotype data were merged with data from Human Genome Diversity Project (HGDP) (Li et al. 2008), 89 Malay individuals from Singapore Genome Variation Project (SGVP) (Teo et al. 2009) and Onge and Jarawa Negritos from Andaman islands were genotyped using Illumina Human 1.2M (SNP population data courtesy of P. Majumder and A. Basu). After merging data sets (supplementary table S1, Supplementary Material online), a total of 291,096 overlapping autosomal SNPs remained for downstream analysis.

### Population Structure Analysis

PCA was used to identify population structure across indigenous Malaysians. PCA analysis was performed on genotyped data of OA combined with Andamanese Negritos, Oceanians, South and East Asian populations in the HGDP, and Malays from SGVP using EIGENSOFT v3.0. To balance sample sizes across our populations, 30 Malay individuals were randomly sampled from SGVP data set (which contains 89 individuals). SNPs with *r*^2^ > 0.5 were pruned out in order to avoid the effects of excessive LD between SNPs. After this pruning a total of 204,426 SNPs remained for analysis. Pairwise Fst distance between populations in same data set were calculated using EIGENSOFT v3.0, and a Neighbor-net tree was constructed by SplitsTree v4 software (Huson and Bryant 2006). ADMIXTURE v1.22, a clustering algorithm, was used on pruned SNPs to estimate the ancestral population clustering (Alexander et al. 2009).

PLINK v1.07 was used to estimate ROH in selected populations. PLINK takes 5,000 kb (50 SNPs) sliding windows across the genome and allows for 1 heterozygous and 5 missing calls in each window. To minimize the effects of LD on ROH, minimum ROH length was set to be 500 kb because it is unusual for LD to extend beyond 500 kb. LD decay for each population was calculated as *r*^2^ using PLINK. Pairwise LD between all possible SNPs was calculated and mean LD was measured in bins of 5 kb.

TreeMix v1.12 (Pickrell and Pritchard 2012) was used to explore the population relationships and migration events. Same data set described above was used to estimate the Maximum Likelihood tree with Yoruba as outgroup. We used blocks of 200 SNPs (-k 200) to account for LD and migration edges added sequentially until the model explained 99% of variances. We estimated the *D* statistics using ADMIXTOOLS (Patterson et al. 2012) to examine gene flow between OAs and surrounding populations. Divergence time between OA and EA was estimated using 399,971 shared SNPs between our data and HapMap 3 (The International HapMap 2005). Effective population size (*N*_e_) and divergence time between OAs and Yoruba in Ibadan (YRI), Han Chinese in Beijing (CHB), and Japanese in Tokyo (JPT) samples were estimated according to the method suggested by McEvoy et al. (2011). To estimate LD, pairwise LD was calculated as *r*^2^ using PLINK v1.07. In order to minimize the effects of small sample size, all individuals were pooled together in their respective OA groups. Admixture time between OAs and EA was estimated by rolloff package using 399,971 SNPs by HapMap3 and OAs.

## Results

**Fig. 2.—:**
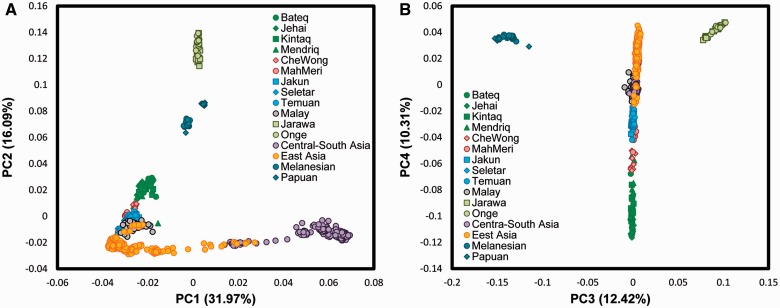
PCA of Orang Aslis and surrounding populations.

To understand population structure across Negritos, other OA subgroups, and their relationship with neighboring populations in Asia and Oceania, a PCA was performed (fig. 2 and supplementary fig. S1, Supplementary Material online). As presented in figure 2*A*, the first component, which captures 32% of total variation, clearly distinguishes South Asian populations from those in the East. From PC2, the Onge and Jarawa, both Negrito subgroups, clustered together and were distinct from other populations. However, they appeared closest to Papuans and Melanesians. The Malaysian Negrito subgroups, while clustering closer to East Asians, showed a tendency toward other Negrito subgroups in Oceania and Andaman islands. The rest of OAs such as Senoi and Proto-Malays as well as Singaporean Malays were located between Malaysian Negritos and East Asian clusters indicating that these groups might be admixed between these two populations. However, both Senoi and Proto-Malay groups lay closer to East Asians on PC4 suggesting that all these populations may have a common origin.

Like PCA analysis, the results of Neighbor-net tree showed that OAs are closest to EA populations. As evident in supplementary figure S2, Supplementary Material online, all four subgroups of Negritos formed a clade, while Senoi and Proto-Malay were positioned at various points between these two clades. The long branches observed in Bateq, Jehai, Kintaq, CheWong, Seletar, and MahMeri suggest strong drift in each of these populations. Interestingly, Seletar located between Malaysian Negritos and Oceanians. The tree also indicated genetic affinity between Andamanese and Oceanians.

**Fig. 3.—:**
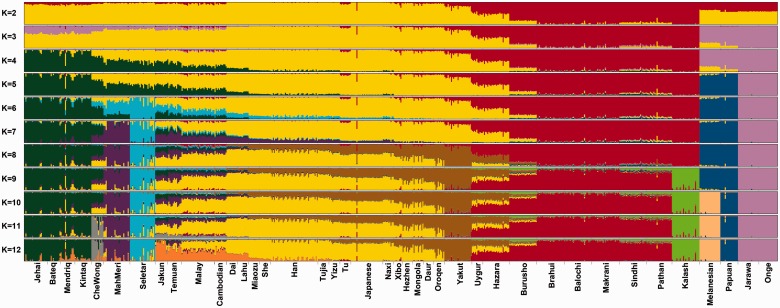
ADMIXTURE analysis of Orang Asli, Andamanese, South Asian, and East Asian ethnic groups from HGDP and Singaporean Malay.

In order to determine critical ancestral components that may have shaped the genetic architecture among the OAs, we applied ADMIXTURE analysis. The results of ADMIXTURE from *K* = 2 to *K* = 12 are shown in figure 3. Each individual is represented as a vertical bar and their corresponding ancestry components are shown by different colors. Different colors indicate different ancestry lineages. As presented, *K* = 2 separated Central-South Asia (red) and EA (yellow) and the latter appears to be the major component in all OA groups. From K = 3, Andamanese component (pink) appeared. This component also presented considerably in Oceanians and in lesser extent in Malaysian Negritos. At higher *K* = 4 and *K* = 5, Negrito (dark green) and Oceanian (dark blue) components appeared respectively. The best model which had the lowest cross validation error suggests nine major ancestral groups which gave rise to the 40 distinct populations included in our study. At *K* = 9, all Negrito subgroups showed similar ancestral patterns. However, we observed small portions of other ancestral components (shown in yellow and purple) in some Negrito individuals (especially Mendriqs).

Results of ADMIXTURE at *K* = 9 also showed that two Senoi subgroups had different ancestral patterns. The purple colored ancestry component is highest in MahMeri, but also present in the Proto-Malay and Malay. The CheWongs appear to have MahMeri, Negrito, and East Asian components. At *K* = 11, CheWong appeared distinct.

Different patterns of ancestry were identified in Proto-Malays. At *K* = 9, Jakun and Temuan had similar ancestral components, but there was a unique substantial component (shown in light blue) only present in the Seletar from *K* = 6. The ADMIXTURE results further support the uniqueness of OAs.

**Fig. 4.—:**
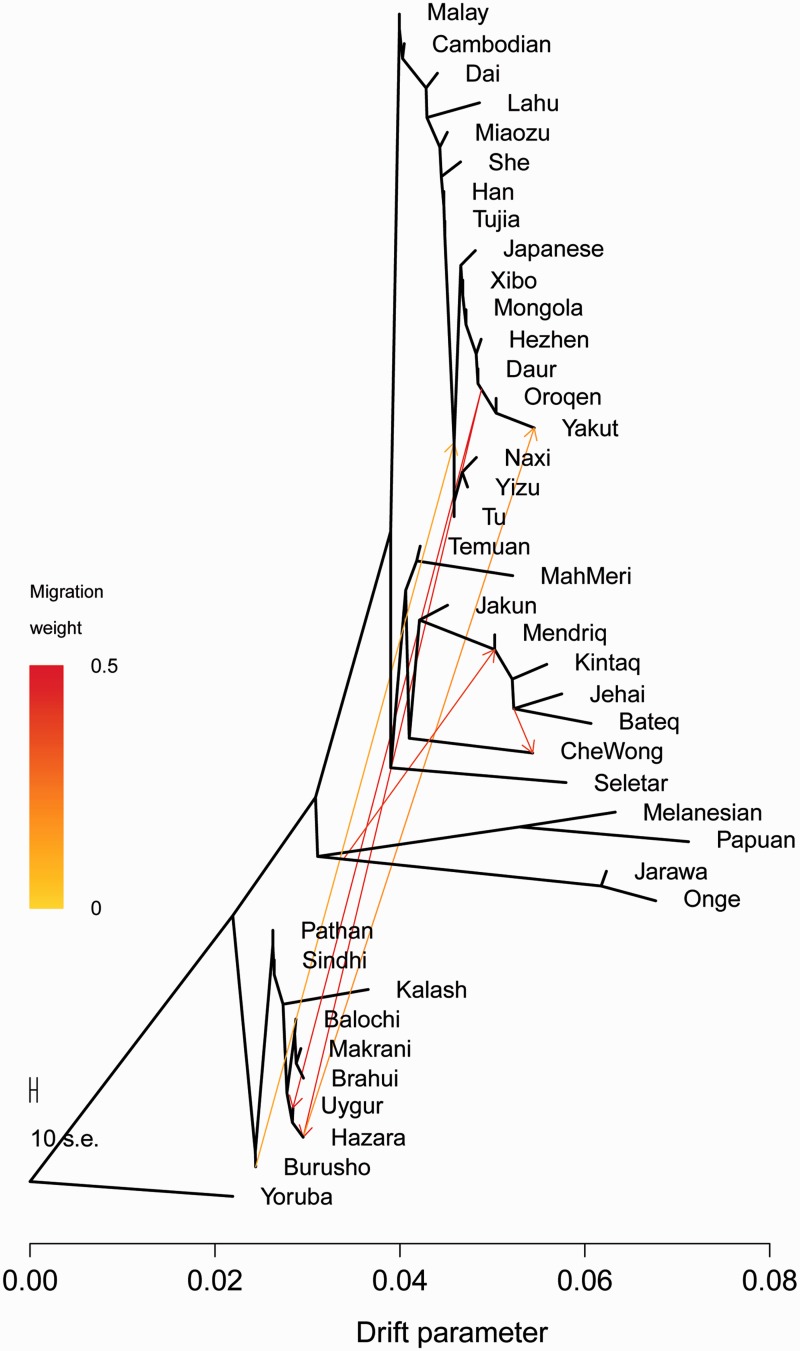
Treemix tree of Orang Asli subgroups, Negrito groups of Andaman Islands, and South and East Asian populations from HGDP.

To understand the relationship between our populations and examine the gene flow between them, we used TreeMix (fig. 4 and supplementary fig. S3, Supplementary Material online). Using Yoruba as root, the graph that best fits our data (99.4% of variances) inferred six migration events. The tree topology was consistent with geographical distribution of populations and with previously shown Neighbor-net tree. Andamanese and Oceanians grouped together in a deep clade, while all OA groups formed a distinct cluster. Focusing on migration events, a migration (migration weight 0.37) directed from root Onge and Jarawa toward Malaysian Negrito root. The resulting tree also highlighted another migration (0.39) from the root of Bateq and Jehai to CheWong.

To further investigate gene flow between OAs and other populations, we used *D* statistics (table 1 and supplementary tables S2 and S3, Supplementary Material online). The computed *D* statistics demonstrated significant gene flow between Andamanese and Malaysian Negritos but there was no significant gene flow detected between Andamanese and other OA groups. This suggests that an earlier gene flow occurred before other OA groups arrived in Peninsular Malaysia. The *D* statistics supported admixture between different OA groups, as gene flows between Negrito/Senoi, Negrito/ Proto-Malays, and Senoi/Proto-Malays were evident. We also traced admixture in AA-speaking OAs and those of Mainland SEA and Lahu and Dai, ethnic groups from South China.

**Table 1:** Computed *D* Statistic Results Showing Gene Flow between Negrito and Other Populations in SEA

Group	*D* Score	*Z* Score^a^	Group	*D* score	*Z* score
D (Jehai, Yoruba; Han, X)			D (Jehai, Yoruba; Japanese, X)		
Temuan	−1.16 × 10^−02^	−10.578	Temuan	−1.82 × 10^−02^	−15.328
Jakun	−1.44 × 10^−02^	−11.125	Jakun	−2.10 × 10^−02^	−15.438
Seletar	−2.00 × 10^−03^	−1.397	Seletar	−8.80 × 10^−03^	−5.923
MahMeri	−9.30 × 10^−03^	−6.931	MahMeri	−1.60 × 10^−02^	−11.455
CheWong	−3.47 × 10^−02^	−21.149	CheWong	−4.11 × 10^−02^	−24.359
Malay	−6.00 × 10^−04^	−0.744	Malay	−7.40 × 10^−03^	−7.999
Cambodian	−2.40 × 10^−03^	−2.464	Cambodian	−9.20 × 10^−03^	−8.482
Lahu	−6.10 × 10^−03^	−5.612	Lahu	−1.30 × 10^−02^	−10.602
Dai	−7.80 × 10^−03^	−8.537	Dai	−1.46 × 10^−02^	−13.701

D (Bateq, Yoruba; Han, X)			D (Bateq, Yoruba; Japanese, X)		
Temuan	−1.02 × 10^−02^	−9.277	Temuan	−1.56 × 10^−02^	−12.926
Jakun	−1.44 × 10^−02^	−10.388	Jakun	−1.98 × 10^−02^	−13.604
Seletar	−1.80 × 10^−03^	−1.25	Seletar	−7.30 × 10^−03^	−4.733
MahMeri	−7.90 × 10^−03^	−5.84	MahMeri	−1.33 × 10^−02^	−9.404
CheWong	−3.63 × 10^−02^	−19.949	CheWong	−4.14 × 10^−02^	−22.355
Malay	2.00 × 10^−04^	0.206	Malay	−5.40 × 10^−03^	−5.496
Cambodian	−1.70 × 10^−03^	−1.635	Cambodian	−7.20 × 10^−03^	−6.351
Lahu	−4.60 × 10^−03^	−4.032	Lahu	−1.02 × 10^−02^	−8.124
Dai	−6.70 × 10^−03^	−7.09	Dai	−1.23 × 10^−02^	−11.212

D (Kintaq, Yoruba; Han, X)			D (Kintaq, Yoruba; Japanese, X)		
Temuan	−1.02 × 10^−02^	−9.504	Temuan	−1.65 × 10^−02^	−14.175
Jakun	−1.26 × 10^−02^	−9.693	Jakun	−1.88 × 10^−02^	−13.683
Seletar	−1.80 × 10^−03^	−1.213	Seletar	−8.10 × 10^−03^	−5.271
MahMeri	−8.20 × 10^−03^	−6.342	MahMeri	−1.44 × 10^−02^	−10.695
CheWong	−3.40 × 10^−02^	−20.837	CheWong	−4.00 × 10^−02^	−23.709
Malay	0.00	−0.056	Malay	−6.40 × 10^−03^	−6.985
Cambodian	−2.00 × 10^−03^	−2.089	Cambodian	−8.30 × 10^−03^	−7.879
Lahu	−5.20 × 10^−03^	−4.788	Lahu	−1.16 × 10^−02^	−9.756
Dai	−7.20 × 10^−03^	−7.749	Dai	−1.36 × 10^−02^	−12.934

^a^Absolute *Z* score >3 shows significant gene flow between populations.

**Fig. 5.—:**
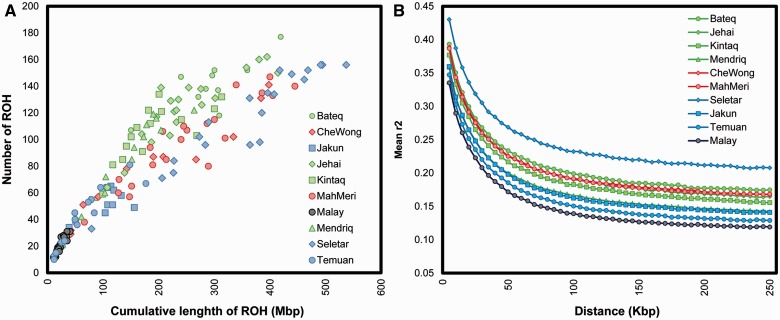
(*A*) Runs of homozygosis in Orang Aslis and Malay from SGVP and (*B*) pattern of linkage disequilibrium decay in Orang Asli groups and SGVP Malay.

Focusing on OAs in Malaysia, we determined inheritance of parental genome components, and calculated ROH in all OA groups against Malay from Singapore. Figure 5*A* shows the distribution of ROH in these populations. As expected, all Negrito groups generally showed long and high ROH compared with other OA groups. This is indicative of small population size or consanguinity. Interestingly, Seletar had the longest ROH among all OA groups which may reflect higher levels of autozygosity.

To further examine the genetic isolation and admixture between OA groups, we calculated pairwise LD between all autosomal SNPs. LD is the nonrandom association of two SNPs and its decay can be affected by factors like drift, admixture, and inbreeding. Figure 5*B* shows the LD decay in OA subgroups and Singaporean Malays. LD in all OA groups was markedly higher even for long pairwise SNPs distances.

We estimated the divergence time (*T*) of OA groups and Africans to be around 67 KYA assuming generation time of 25 years which is a good agreement with other reported estimations of EA and African divergence previously (McEvoy et al. 2011; Pugach et al. 2013). Our results inferred earlier divergence of Negritos from EA in 14–15 KYA which predate those of Senoi (10–11 KYA) and Proto-Malay (8–9 KYA) (table 2). Admixture time estimation between OA groups using “rolloff” showed that the admixture date between Negrito and Senoi to be around 40 generations which was older than Negrito/Proto-Malay and Senoi/Proto-Malay admixture which occurred around 20 generations before the present.

**Table 2:** Divergence Time (KYA) Estimation between OA Groups and YRI, CHB, and JPT

	YRI	CHB	JPT
Negrito	66.8	14.5	14.6
Senoi	67.5	10	11
Proto-Malay	66.9	8.2	9.2
YRI	—	72	72

## Discussion

Despite the rich ethnic diversity present in SEA, the region has been underrepresented in large-scale international genome data sets such as HAPMAP and 1000 Genome Project (Lu and Xu 2013). Diverse linguistic, morphological, and anthropological characteristics found in minor ethnic groups of Malaysia, known as OA, offered a promising opportunity to understand the populations of East Asia and SEA.

Our investigation has contributed substantially more data and provided more comprehensive insight into the population structure of diverse indigenous groups and their prehistoric links to other populations in mainland SEA and East Asia. Apparently, the OAs are genetically closer to EA populations compared with those in South Asia or Oceania. However, our results provided evidences supporting genetic affinity between Malaysian and Andamanese Negritos. Our results are entirely consistent with other SNP studies suggesting link between Andamanes, Malaysian Negritos, and Melanesians (Reich et al. 2011; Chaubey and Endicott 2013).

On a finer scale, Malaysia Negrito subgroups were clearly different from EA populations. This distinct pattern may have resulted from genetic drift. It is also conceivable that they had longer periods of isolation from other inhabitants in the region, as indicated by Fst and LD decay. The ancestral component (dark green) “belonging” to Malaysian Negritos was also spread among Southeast Asian and Southern Chinese populations. However, although Negritos predominantly shared this ancestral component, the Mendriq shared more portions of other ancestral components with East Asians and Senoi. This suggests more recent gene flow between them and their neighboring populations, most likely Malays. A similar observation was reported in Jehai, a Negrito subgroup using a less SNP (Jinam et al. 2013).

The Senoi and Proto-Malay were closely related to EA, either because they share relatively recent common ancestors or because of recent gene flow. However, different patterns emerged in Seletar and CheWong. The corresponding ancestral component of Seletar, a subgroup of Proto-Malay, emerged at *K* = 6 in ADMIXTURE and Neighbor-net tree showed an affinity to the Oceanian. Anthropological information regarding origins of the Seletar is scarce and anecdotal. There is a paucity of information about this community. It is plausible that Seletar might have experienced a recent bottleneck as suggested by the long stretches of LD in their genome. The low levels of mtDNA diversity (Jinam et al. 2012) also provide support for the likelihood of a bottleneck in this population. ADMIXTURE and TreeMix results from CheWong suggest that they are intermediate between Negritos and Senois. Because CheWong appeared distinct at *K* = 11, it can be inferred that their ancestors experienced one or possibly more admixture events in the past, and later became isolated from founding populations. The argument for CheWongs to be admixed is supported by several factors. First, the cultural practices of CheWong are more similar to other Senoi rather than Negritos, while their language is northern Aslian, similar Negrito dialects. Physically, they appear to have intermediate phenotypes between Negrito and Senoi. The genetic evidence presented here for the first time may reduce disagreement among various anthropologists who study tribes in SEA (Benjamin 2013).

The extent of ROH which are identical segments of an individual’s genome inherited from each parent may be indicative of historical events such as bottlenecks, isolation, and consanguinity within populations. Our findings of markedly longer ROH in Negritos, who are the smallest OA group and fast dwindling, may be due to their small population size and isolation after an early divergence. Given that marriages between siblings and cousins are generally prohibited in current Negrito communities, inbreeding is unlikely to have occurred, although we cannot discount this entirely (Benjamin 2013). They traditionally live in small groups composed of few families; so maintaining a small population over time may have resulted in enriched ROH among them. This parallels some African forager communities that have same lifestyle as hunter-gatherer Negritos (Petersen et al. 2013; Patin et al. 2014).

The longest ROH observed in Seletar may best be explained by the occurrence of a population bottleneck. In contrast, other Proto-Malay groups had shorter and fewer ROH compared with Seletar reflecting their larger outbred communities. LD in Negritos was generally higher compared with other OA groups, a likely consequence of their isolation. The LD patterns from our results are similar to those reported for other isolated groups in Africa and Europe (Gross et al. 2011; Esko et al. 2013; Patin et al. 2014).

The Negrito divergence time is consistent with archeological findings regarding the advent of Hoabinhian culture in Mainland SEA (Bellwood 2007). The genetic evidence supports the view that Malaysian Negritos are descendants of Hoabinhian hunter-gatherers who occupied northern parts of Peninsular Malaysia during late Pleistocene. These hunter-gatherers later interacted with Senoi agriculturists during early Holocene era. It may have been these agriculturists who may have introduced AA-based Aslian languages to Negritos. This time frame also coincides with the Early Train migrations from north to south approximately 10–30 KYA (Jinam et al. 2012). However, our time estimation on LD decay can be affected by any bottleneck experienced by these groups. It has been shown that bottlenecks may result in overestimations of LD in populations which consequently result in underestimation of Ne and divergence time. Nevertheless, there are some challenges associated with our investigation. The ascertainment bias that may be present may affect LD estimation. The considerable difference between Negrito/Senoi and Negrito/Proto-Malay admixture date may suggest that the migration of Senoi ancestors to the Malaysian peninsular occurred earlier than those of Proto-Malays. The latter are believed to be a part of Out-of-Taiwan Austronesian expansion. However, our admixture time estimation seems to be much earlier than archeological reports. In the absence of better analytical methods, our analysis relied on rolloff which may reflect only the most recent admixture event, rather than anything earlier.

To circumvent inaccuracy and further refine divergence times, we performed *D* statistics to trace ancient admixture within different OA groups and between OAs and other populations in EA. Interestingly, we report gene flow between AA-speaking OAs and Mainland Southeast Asia (MSEA) and Southern Chinese populations. Existence of Negrito ancestral components in some MSEA has been reported by previous studies (HUGO Pan-Asia SNP Consortium 2009).

In summary, we have demonstrated that the current OA while related, are genetically distinct. The Negritos are very different both phenotypically and genetically. The detailed results we have obtained lead us to speculate that their ancestors contributed significant ancestral genetic components probably during the late Pleistocene to the populations of East Asia and SEA. The continuum in divergence times from Negritos to Senois to Proto Malays coupled with the language transitions provide support to a narrative of at least three major human migrations starting with Out of Africa, then the Early Train followed by Out-of-Taiwan Austronesian expansion.

## Supplementary Material

Supplementary figures S1–S3 and tables S1–S3 are available at *Genome Biology and Evolution* online (http://www.gbe.oxfordjournals.org/).

Supplementary Data
