# Supplementary material for: Unravelling the Genetic History of Negritos and Indigenous Populations of Southeast Asia
Source: Genome Biol Evol. 2015 Apr 14;7(5):1206–15. doi: 10.1093/gbe/evv065 (PMC4453060; doi:10.1093/gbe/evv065)
Supplement: Supplementary Data [file supp_7_5_1206__index.html]

Unravelling the Genetic History of Negritos and Indigenous populations of Southeast Asia — Unravelling the Genetic History of Negritos and Indigenous Populations of Southeast Asia — Supplementary Data 

# Unravelling the Genetic History of Negritos and Indigenous Populations of Southeast Asia

## Supplementary Data

files

**Files in this Data Supplement:**

- Supplementary Data - docx file
- Supplementary Data - docx file
