# Supplementary material for: Unravelling the Genetic History of Negritos and Indigenous Populations of Southeast Asia
Source: Genome Biol Evol. 2015 Apr 14;7(5):1206–15. doi: 10.1093/gbe/evv065 (PMC4453060; doi:10.1093/gbe/evv065)
Supplement: Supplementary Data [file supp_evv065_Suppl_data.docx]

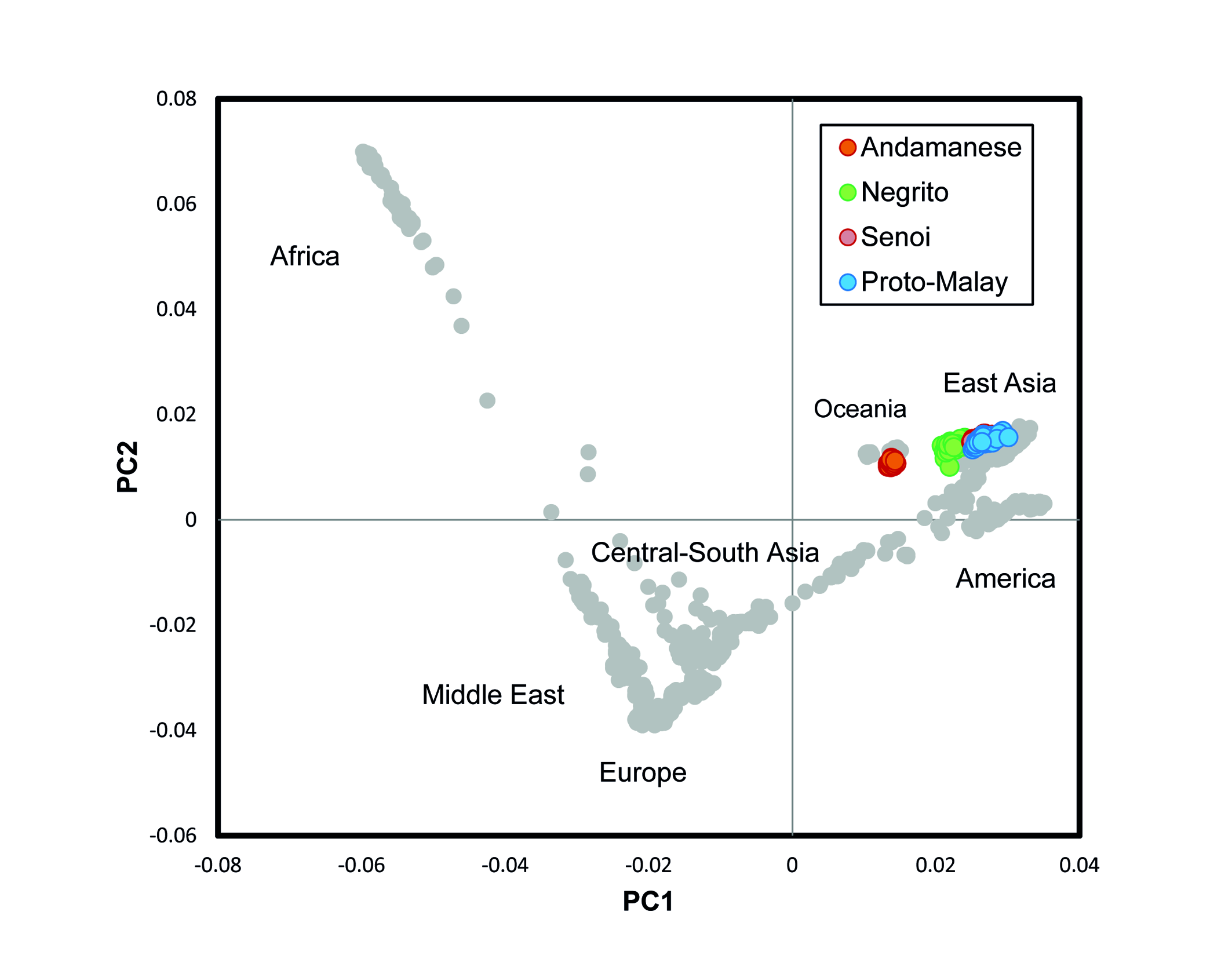


Fig. S1- Principal Component analysis of Orang Asli and Andamanese (color) with populations in HGDP (grey).


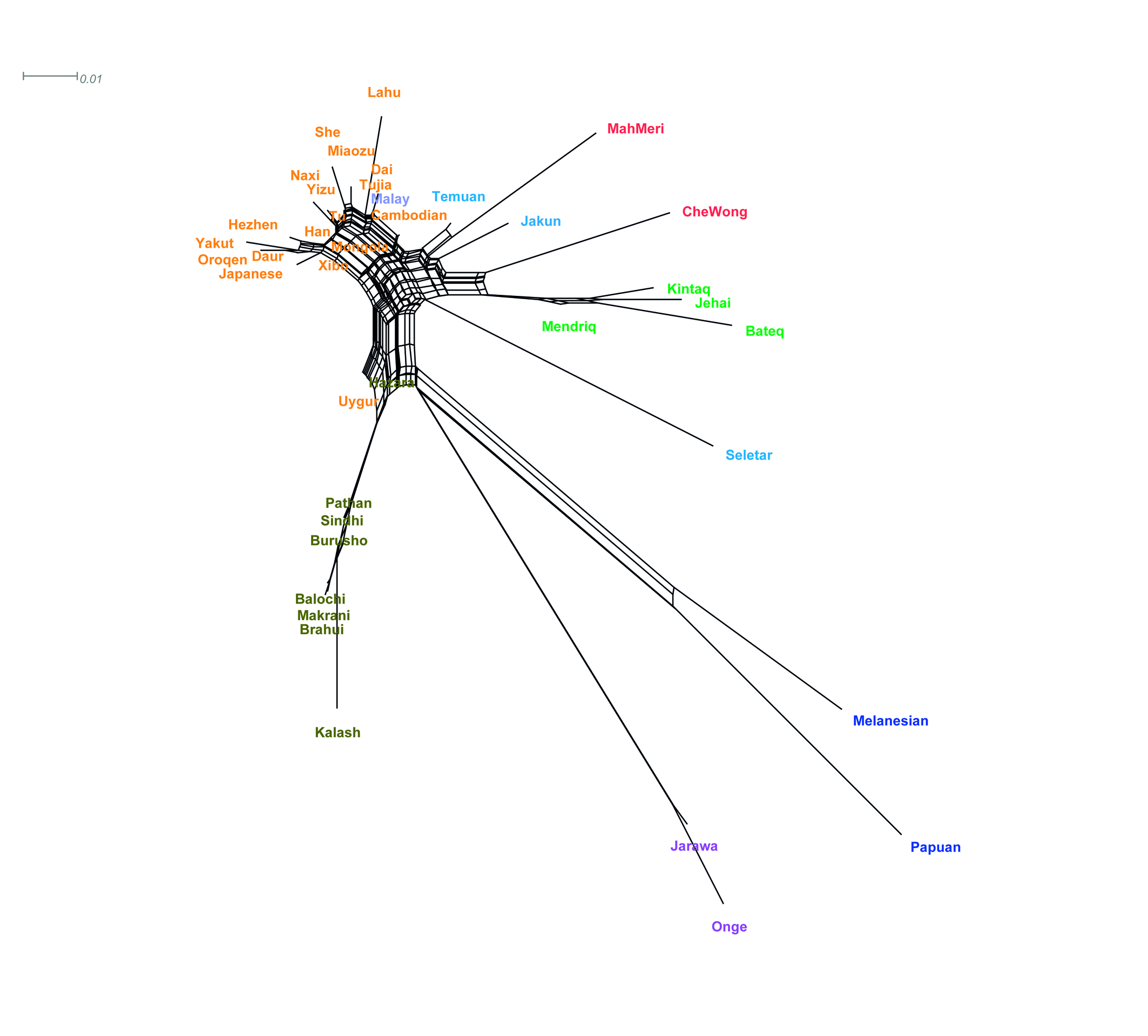


Fig. S2- Neighbor-Net tree of Orang Asli, Andamanese, South and East Asian ethnic groups from HGDP.


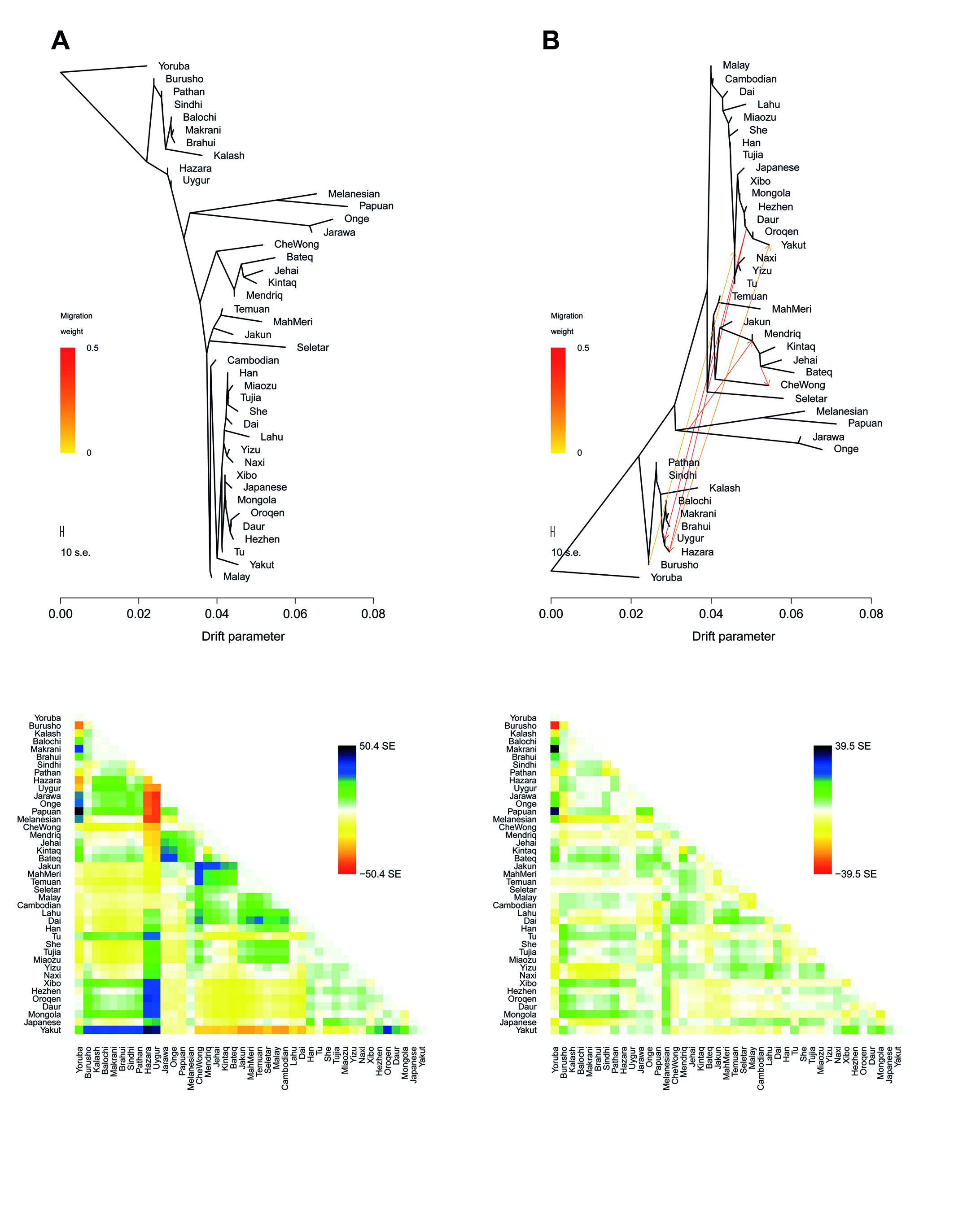
Fig. S3- (A) Treemix analysis of Orang Aslis and neighboring populations. A) Maximum likelihood tree and residuals plot, B) model with 6 gene flow events.

Table S1- Summary of populations used in this study

| Population | Country | N | Data Set |  | Population | Country | N | Data Set |
| --- | --- | --- | --- | --- | --- | --- | --- | --- |
| Bateq | Malaysia | 12 | This study |  | Uygur | China | 10 | HGDP |
| Jehai | Malaysia | 22 | This study |  | Cambodian | Cambodia | 11 | HGDP |
| Kintaq | Malaysia | 15 | This study |  | Dai | China | 10 | HGDP |
| Mendriq | Malaysia | 13 | This study |  | Daur | China | 9 | HGDP |
| MahMeri | Malaysia | 24 | This study |  | Han | China | 44 | HGDP |
| CheWong | Malaysia | 11 | This study |  | Hezhen | China | 9 | HGDP |
| Seletar | Malaysia | 23 | This study |  | Japanese | Japan | 29 | HGDP |
| Temuan | Malaysia | 13 | This study |  | Lahu | China | 10 | HGDP |
| Jakun | Malaysia | 11 | This study |  | Miaozu | China | 10 | HGDP |
| CHB | China | 84 | HapMap Phase 3 |  | Mongola | China | 10 | HGDP |
| JPT | Japan | 86 | HapMap Phase 3 |  | Naxi | China | 10 | HGDP |
| YRI | Nigeria | 113 | HapMap Phase 3 |  | Oroqen | China | 10 | HGDP |
| Onge | India | 17 | A.Basu & P Majumder |  | She | China | 10 | HGDP |
| Jarawa | India | 19 | A.Basu & P Majumder |  | Tu | China | 10 | HGDP |
| Balochi | Pakistan | 25 | HGDP |  | Tujia | China | 10 | HGDP |
| Brahui | Pakistan | 25 | HGDP |  | Xibo | China | 8 | HGDP |
| Burusho | Pakistan | 25 | HGDP |  | Yakut | Siberia | 25 | HGDP |
| Hazara | Pakistan | 24 | HGDP |  | Yizu | China | 10 | HGDP |
| Kalash | Pakistan | 25 | HGDP |  | Melanesian | Bougainville | 19 | HGDP |
| Makrani | Pakistan | 25 | HGDP |  | Papuan | Papua New Guinea | 16 | HGDP |
| Pathan | Pakistan | 23 | HGDP |  | Yoruba | Nigeria | 24 | HGDP |
| Sindhi | Pakistan | 25 | HGDP |  | Malay | Singapore | 89 | SGVP |

Table S2- Dstat analysis shows gene flow between Andamanese and Malaysian Negritos.

| Group | D score | Z score* | Group | D score | Z score |
| --- | --- | --- | --- | --- | --- |
| D(Onge, Yoruba; Han, X) |  |  | D(Onge, Yoruba; Japanese, X) |  |  |
| Bateq | -7.00×10^-03^ | -4.103 | Bateq | -7.60×10^-03^ | -4.283 |
| Jehai | -7.20×10^-03^ | -4.778 | Jehai | -7.80×10^-03^ | -4.933 |
| Kintaq | -6.00×10^-03^ | -4.113 | Kintaq | -6.60×10^-03^ | -4.230 |
| Mendriq | -5.60×10^-03^ | -4.092 | Mendriq | -6.20×10^-03^ | -4.286 |
| Temuan | -1.80×10^-03^ | -1.544 | Temuan | -2.40×10^-03^ | -1.906 |
| Jakun | -2.70×10^-03^ | -2.055 | Jakun | -3.30×10^-03^ | -2.338 |
| Seletar | -3.00×10^-04^ | -0.164 | Seletar | -9.00×10^-04^ | -0.57 |
| MahMeri | -2.80×10^-03^ | -2.089 | MahMeri | -3.50×10^-03^ | -2.364 |
| CheWong | -4.50×10^-03^ | -2.827 | CheWong | -5.10×10^-03^ | -3.033 |
| D(Jarawa, Yoruba; Han, X) |  |  | D(Jarawa, Yoruba; Japanese, X) |  |  |
| Bateq | -6.70×10^-03^ | -4.094 | Bateq | -7.50×10^-03^ | -4.402 |
| Jehai | -7.70×10^-03^ | -5.183 | Jehai | -8.40×10^-03^ | -5.467 |
| Kintaq | -6.40×10^-03^ | -4.49 | Kintaq | -7.20×10^-03^ | -4.742 |
| Mendriq | -6.10×10^-03^ | -4.561 | Mendriq | -6.90×10^-03^ | -4.847 |
| Temuan | -1.60×10^-03^ | -1.447 | Temuan | -2.40×10^-03^ | -1.979 |
| Jakun | -3.10×10^-03^ | -2.422 | Jakun | -3.90×10^-03^ | -2.824 |
| Seletar | -6.00×10^-04^ | -0.387 | Seletar | -1.40×10^-03^ | -0.879 |
| MahMeri | -2.90×10^-03^ | -2.171 | MahMeri | -3.70×10^-03^ | -2.589 |
| CheWong | -4.90×10^-03^ | -3.191 | CheWong | -5.70×10^-03^ | -3.481 |

* Absolute Z score >3 shows significant gene flow between populations

Table S3- Dstat shows gene flow between Senois and SEA populations.

| Group | D score | Z score* | Group | D score | | Z score |
| --- | --- | --- | --- | --- | --- | --- |
| D(MahMeri, Yoruba; Han, X) |  |  | D(MahMeri, Yoruba; Japanese, X) | |  |  |
| Temuan | -2.17×10^-02^ | -18.494 | Temuan | -3.17×10^-02^ | | -25.313 |
| Jakun | -4.50×10^-03^ | -3.296 | Jakun | -1.46×10^-02^ | | -9.939 |
| Seletar | 3.40×10^-03^ | 2.17 | Seletar | -6.90×10^-03^ | | -4.219 |
| Malay | 8.00×10^-04^ | 0.955 | Malay | -9.50×10^-03^ | | -9.774 |
| Cambodian | -1.40×10^-03^ | -1.48 | Cambodian | -1.17×10^-02^ | | -10.455 |
| Lahu | -6.30×10^-03^ | -5.683 | Lahu | -1.66×10^-02^ | | -13.828 |
| Dai | -1.05×10^-03^ | -11.457 | Dai | -2.09×10^-02^ | | -18.877 |
| D(CheWong, Yoruba; Han, X) |  |  | D(CheWong, Yoruba; Japanese, X) | |  |  |
| Temuan | -1.19×10^-02^ | -10.105 | Temuan | -2.09×10^-02^ | | -16.751 |
| Jakun | -1.21×10^-02^ | -8.777 | Jakun | -2.10×10^-02^ | | -14.573 |
| Seletar | 1.60×10^-03^ | 0.992 | Seletar | -7.50×10^-03^ | | -4.545 |
| Malay | -6.00×10^-04^ | -0.737 | Malay | -9.70×10^-03^ | | -9.929 |
| Cambodian | -2.80×10^-03^ | -2.731 | Cambodian | -1.19×10^-02^ | | -10.557 |
| Lahu | -7.30×10^-03^ | -6.199 | Lahu | -1.65×10^-02^ | | -13.015 |
| Dai | -9.80×10^-03^ | -10.122 | Dai | -1.90×10^-02^ | | -16.664 |

* Absolute Z score >3 shows significant gene flow between populations
