# Supplementary material for: Unravelling the Genetic History of Negritos and Indigenous Populations of Southeast Asia
Source: Genome Biol Evol. 2015 Apr 14;7(5):1206–15. doi: 10.1093/gbe/evv065 (PMC4453060; doi:10.1093/gbe/evv065)
Supplement: Supplementary Data [file supp_evv065_Suppl_file.docx]

**Supplementary Materials:**

**Figures:**

Fig. S1- Principal Component analysis of Orang Asli and Andamanese (color) with populations in HGDP (grey).

Fig. S2- Neighbor-Net tree of Orang Asli, Andamanese, South and East Asian ethnic groups from HGDP.

Fig. S3- (A) Treemix analysis of Orang Aslis and neighboring populations. A) Maximum likelihood tree and residuals plot, B) model with 6 gene flow events.

**Tables:**

Table S1- Summary of populations used in this study

Table S2- Dstat analysis shows gene flow between Andamanese and Malaysian Negritos.

Table S3- Dstat shows gene flow between Senois and SEA populations.
